# Paraspinal and Presacral Extramedullary Hematopoiesis: A Rare Manifestation of Polycythemia Vera

**DOI:** 10.5812/iranjradiol.4297

**Published:** 2013-08-30

**Authors:** Kaleem Ahmad, Sajid Ansari, Roshan Koirala, Meenu Agarwal, Shatdal Chaudhary

**Affiliations:** 1 Department of Radiodiagnosis, B.P. Koirala Institute of Health Sciences, Dharan, Nepal; 2 Department of Radiodiagnosis, Nobel Medical College, Nepal; 3 Department of Pathology, B.P. Koirala Institute of Health Sciences, Dharan, Nepal; 4 Department of Medicine, B.P. Koirala Institute of Health Sciences, Dharan, Nepal

**Keywords:** Hematopoiesis, Extramedullary, Polycythemia Vera

## Abstract

Extramedullary hematopoiesis is characterized by the presence of hematopoietic tissue outside the bone marrow. Extrathoracic extramedullary hematopoiesis is a rare and usually asymptomatic condition. We report a case of a 38-year-old female with paraspinal and presacral extramedullary hematopoiesis with polycythemia vera. Clinical and laboratory evaluation, along with radiological and histopathological findings are described. The diagnosis of the disease was confirmed by CT-guided biopsy. Review of literature is presented.

## 1. Introduction

Polycythemia vera is a myeloproliferative disorder of clonal origin arising at the level of pluripotential hematopoietic stem cell and resulting in neoplastic proliferation of erythroid, myeloid and megakaryocytic elements in the bone marrow, an increased red blood cell mass and usually raised blood counts of the three major hematopoietic cell lines (1). Extramedullary hematopoiesis (EMH) is a rare disorder and is found in patients with prolonged anemias such as hemolytic anemias, myeloproliferative disorders and some neoplasms as a compensatory mechanism that is needed for sufficient erythrogenesis (2). It is rarely encountered in the thorax and extrathoracic locations i.e. paraspinal (lumbar) and presacral regions are even less common (3, 4). We report a case of paraspinal (lumbar) and presacral EMH in a female with polycythemia vera.

## 2. Case Presentation

A 38-year-old female presented in the Department of Radiodiagnosis with pain in the left upper quadrant of the abdomen for 3 months. The patient was a diagnosed case of polycythemia vera with a history of multiple phlebotomies in the past. On examination, she was afebrile and her pulse rate, respiratory rate and blood pressure were within normal limit. There was no evidence of lymphadenopathy, cyanosis, pallor, icterus, clubbing or any other significant relevant history. Her sensory and motor functions were normal. Abdominal examination revealed a moderate splenomegaly of 7 cm below the left costal margin.

Laboratory data are mentioned in Table 1. The patient had an elevated total leukocyte count, hemoglobin and packed cell volume. The clinical impression was polycyhtemia vera with splenomegaly. Arterial blood gas (ABG) was also normal. Plain radiograph of the chest was normal. 

**Figure 1.**
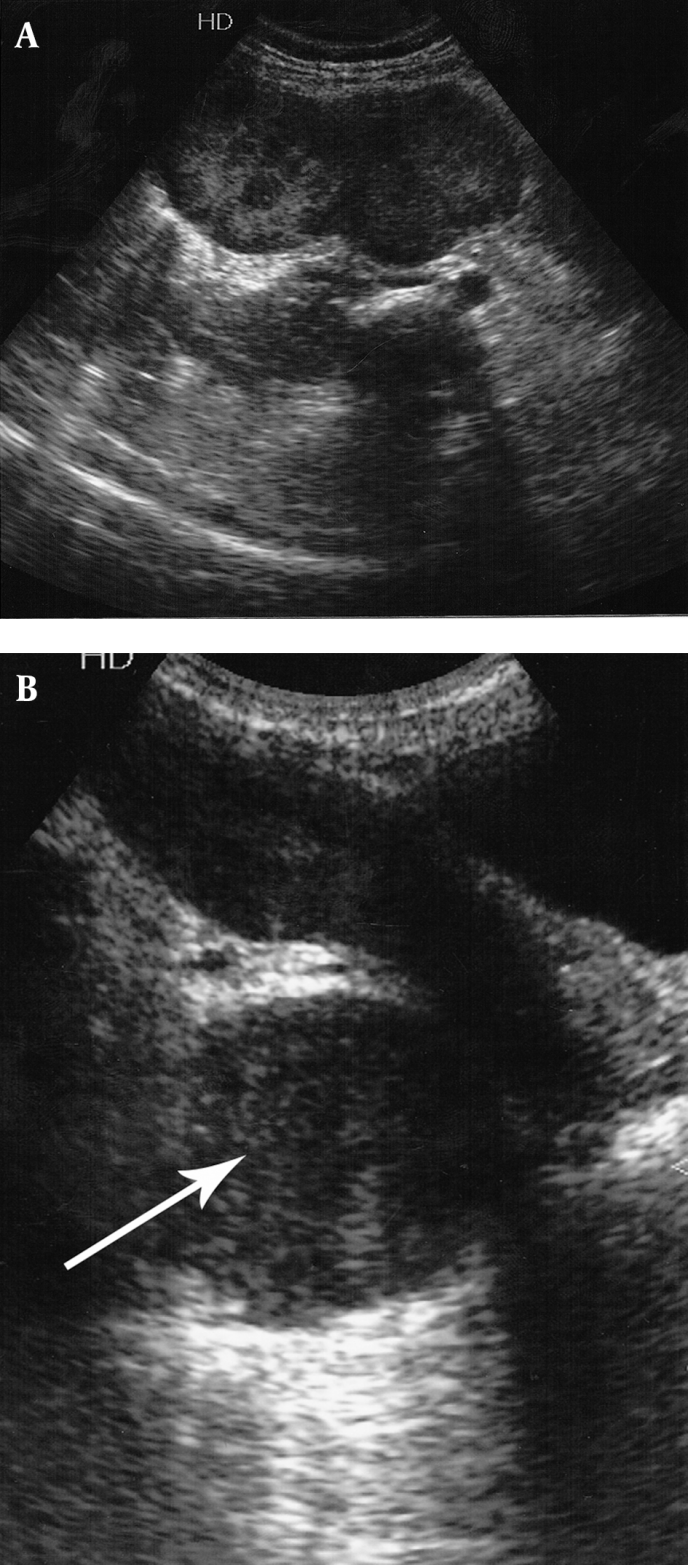
Ultrasonography of the abdomen and pelvis demonstrating well-defined hypoechoic lesions in the A, right paraspinal and B, the presacral regions.

Ultrasonography (USG) of the abdomen and pelvis demonstrated moderate splenomegaly along with hypoechoic lesions sized 7.5×4 cm and 5.5×3.5 cm in the right paraspinal (lumbar) and presacral regions, respectively (Figure 1). 

**Table 1.** Laboratory Findings of the Patient

Laboratory investigations	Findings
**Hemoglobin**	19.8 gm%
**Total leukocyte count**	23,000/cu mm^3^
**Differential count**	Polymorphonuclears- 91%, Lymphocytes 8%, Eosinophils 1%
**Packed cell volume**	65.3%
**Mean cell volume**	73 fl
**Reticulocyte count**	7.0%
**ESR**	25 mm in the first hour
**Total bilirubin**	0.8 mg/dl
**Conjugated bilirubin**	0.2 mg/dl
**SGOT**	27 U/L
**SGPT**	37 U/L
**Alkaline phosphatase**	199 U/L
**Total protein, albumin, uric acid, serum creatinine and blood glucose levels**	Within normal limits

**Figure 2.**
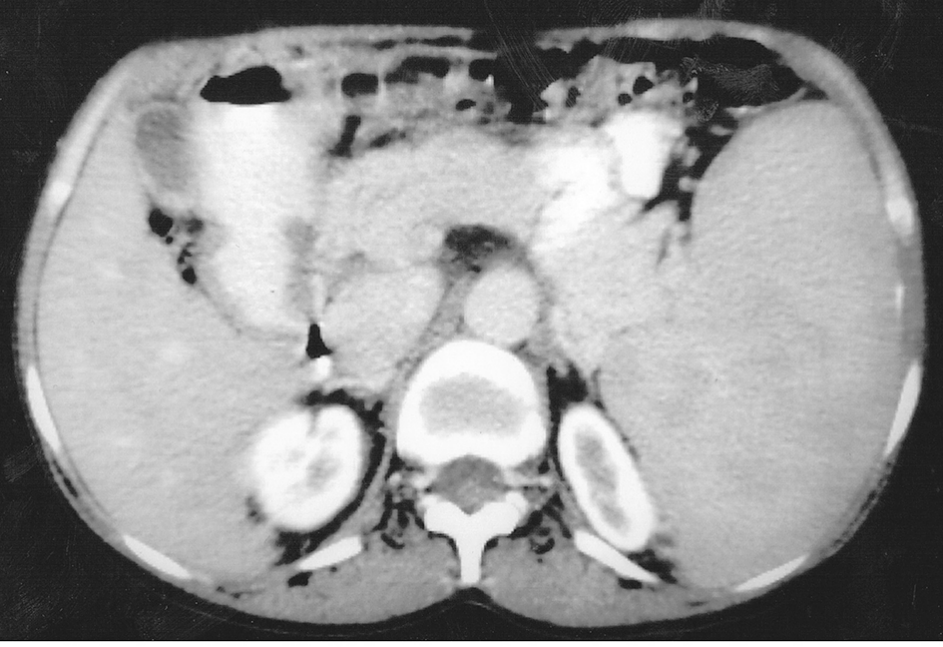
Abdominopelvic MDCT; axial sections showing moderate splenomegaly.

**Figure 3.**
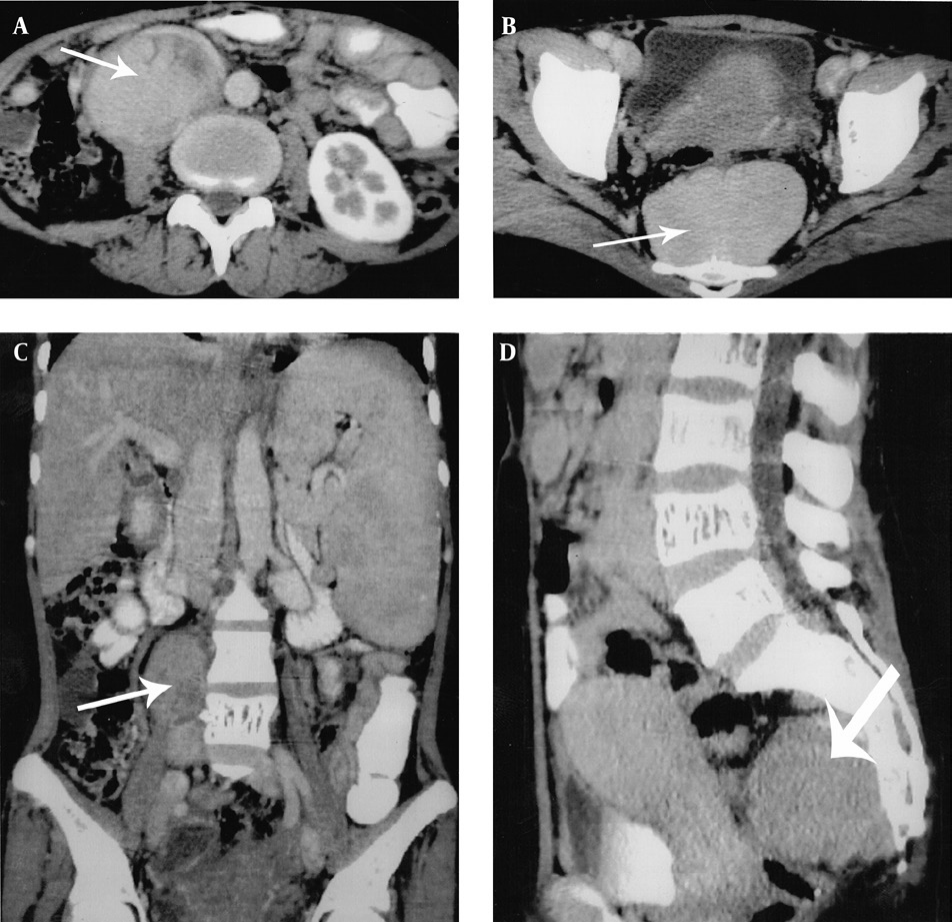
Abdominopelvic MDCT; axial sections show intensely enhancing soft tissue lesions in the right paraspinal region (A) and the presacral region (B). Coronal reformatted CT image (C) shows moderate splenomegaly and intensely enhancing soft tissue lesion in the right paraspinal region. Sagittal reformatted CT image (D) shows intensely enhancing soft tissue lesion in the presacral region.

Multidetector computed tomography (MDCT) of the abdomen and pelvis were performed from the level of the diaphragm to the coccyx. Non-contrast CT followed by contrast-enhanced CT was done by taking 10×10 mm axial sections. Images were reconstructed in thin sections (1.25 mm) in the coronal plane. MDCT revealed moderate splenomegaly (Figure 2) and an intensely enhancing well-defined 8×4 cm sized lesion of soft tissue attenuation in the right paraspinal (lumbar) region at the level of L2-L3 vertebrae (Figure 3 A and B). Another intensely enhancing 6×4.5 cm sized similar lesion of soft tissue attenuation was also seen in the presacral region causing mild scalloping of the sacrum (Figure 3 C and D). 

**Figure 4.**
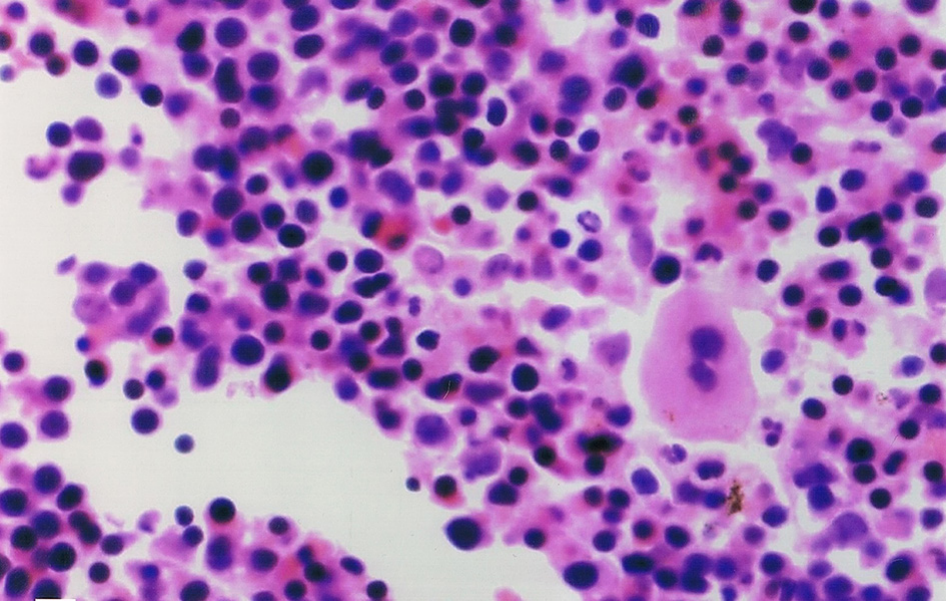
Histopathologic view of the tissue sample taken in CT-guided biopsy showing hematopoietic tissues consistent with extramedullary hematopoiesis.

CT-guided biopsy from the right paraspinal (lumbar) and presacral masses revealed hematopoietic tissue containing myeloid, erythroid and megakaryocytic lines showing maturation; an excess of immature cells was present in some microscopic fields (Figure 4). Correlating the clinical, laboratory, radiological and histopathological findings, the final diagnosis of polycythemia vera with extramedullary hematopoiesis presenting as paraspinal (lumbar) and presacral masses was made. 

## 3. Discussion

EMH is a physiological compensatory mechanism in which there is formation of normal blood cells outside the bone marrow because the bone marrow isunable to meet the normal circulatory demands (5). EMH is seen in various hematological disorders such as myelofibrosis, polycythemia vera, lymphoma, leukemia or post-bone marrow irradiation (6, 7). There are various sites in the body that are involved in EMH like the spleen, liver, thymus, breast, lung pleura, heart, kidneys, prostate, suprarenal glands, ovaries, intestine, sclera, lymph nodes, retroperitoneal soft tissues, skin, peripheral and cranial nerves, and the spinal canal (8-12). Active hematopoiesis occurs at these sites during the period of gestation that stops at birth.However, if there is prolonged ineffective erythropoiesis, the extramedullary hematopoietic tissues retain its ability to produce red cells (13). According to some authors, extramedullary hematopoietic tissue can be extruded through the trabecular bone of the vertebral body or through the thinned trabeculae at the proximal ends of the ribs (14). During the early phase of its evolution, immature and mature cells (predominantly erythroid and myeloid series) and dilated sinusoids containing precursors of red cells are found at the site of paraspinal EMH and later, the lesions become inactive revealing some fatty tissue, fibrosis or massive iron deposits (15). EMH is associated with chronic anemic states that is most commonly seen in cases of thalassemia; however, it is less common in other anemic and myeloproliferative disorders such as myelofibrosis and polycythemia vera (6). Rarely, it can cause cord compression, pleural effusion, massive hemothorax and respiratory failure. Intrathoracic and extrathoracic EMH is a rare cause of paraspinal mass that should be differentiated from other common causes, such as neurogenic tumors, lymphoma, metastasis, paravertebral abscess, and lateral meningocele (16). A paraspinal location for the hematopoietic tissue occurs in 11-15% of cases with EMH (8). Approximately 80% of these cases are asymptomatic that are usually diagnosed incidentally at imaging. (17). Paraspinal EMH can usually be seen as an isolated mediastinal mass or it can be found in combination with abdominal paraspinal masses (16). As these masses are highly vascular in nature, on contrast enhanced CT scans, it shows intense homogeneous enhancement (18). Reports on EMH in polycythemia rubra vera (PRV) are mentioned in Table 2. Most of the studies have shown masses in the cervical and thoracic regions, but two of the studies have discussed the mass in the lumbar paravertebral region. MacCallum et al.(19) studied a case of diagnosed polycythemia vera in the proliferative phase in a 52-year-old man who developed EMH in cervical and lumbar paravertebral regions and died as a result of cervical cord compression. But in our study, the masses are in lumbar paravertebral and presacral regions with no evidence of compression of the cord. Masses in lumbar paravertebral and presacral regions in EMH are rarely reported. 

**Table 2.** Previous Case Reports on EMH in Polycythemia Rubra Vera

Authors	Year	Patient Age (yr)	Patient Gender	PRV	EMH
**Oustwani et al.**(20)	1980	70	M	Present	Present
**Rice et al.**(21)	1980	68	M	Present	Present
**MacCallum et al.**(19)	1988	52	M	Present	Present
**Jackson et al.**(7)	1989	52	M	Present	Present
**de Morais et al.**(6)	1996	46	M	Present	Present
**Ohta et al.** (22)	2002	59	F	Present	Present
**Masmas et al.** (23)	2003	30	M	Present	Present
**Haran and Ni **(24)	2003	68	M	Present	Present
**Scott and Poynton** (25)	2008	69	M	Present	Present

The signal intensity of EMH on MRI depends on the activity of the hematopoietic cells (13). In active hematopoiesis, the lesions show intermediate signal intensity on T1weighted images and high signal intensity on T2 weighted images representing either immature or mature erythroid and myeloid series. In chronic inactive hematopoiesis, the lesions seem hypointense both on T1 and T2 weighted images due to iron deposition, or they show high signal intensity when there is predominance of fatty tissues. After administration of gadolinium, the active hemotopoietic lesions show intense enhancement while there is no enhancement of the hematopoietic lesions in the inactive stage of EMH (26). MRI is currently the gold standard for demonstrating spinal EMH; it produces superior soft tissue delineation and is highly sensitive (27).

Polycythemia vera is treated with alkylating agents, radioactive phosphorus (32P) and venesection. If there is compressive myelopathy, laminectomy is done. It commonly transforms from a proliferative into myelofibrotic stage. After treatment with alkylating agents or radioactive phosphorus (32P), upto 15% of cases of polycythemia vera can be transformed into acute myeloblastic leukemia (AML) (28, 29). In our case, the patient is a known case of polycythemia vera; the presence of paraspinal and presacral masses in the lumbar and presacral regions were supposed to be EMH, but few cases of polycythemia vera may transform into AML and granulocytic sarcoma, so biopsy was done to confirm EMH and to rule out the above mentioned entities.

In conclusion, based on characteristic clinical, biochemical, radiological and histopathological findings, it is important to consider the possibility of extramedullary hematopoiesis in the differential diagnosis of paraspinal (lumbar) and presacral masses in polycythemia vera.
